# Texture Features of Proton Density Fat Fraction Maps from Chemical Shift Encoding-Based MRI Predict Paraspinal Muscle Strength

**DOI:** 10.3390/diagnostics11020239

**Published:** 2021-02-04

**Authors:** Michael Dieckmeyer, Stephanie Inhuber, Sarah Schlaeger, Dominik Weidlich, Muthu Rama Krishnan Mookiah, Karupppasamy Subburaj, Egon Burian, Nico Sollmann, Jan S. Kirschke, Dimitrios C. Karampinos, Thomas Baum

**Affiliations:** 1 Department of Diagnostic and Interventional Neuroradiology, Klinikum rechts der Isar der Technischen Universitär München, Ismaninger 22, 81675 Munich, Germany; sarah.schlaeger@tum.de (S.S.); egon.burian@tum.de (E.B.); nico.sollmann@tum.de (N.S.); jan.kirschke@tum.de (J.S.K.); thomas.baum@tum.de (T.B.); 2 Department of Sport and Health Sciences, Technical University of Munich, Georg-Brauchle-Ring 60, 80992 Munich, Germany; stephanie.inhuber@tum.de; 3 Department of Diagnostic and Interventional Radiology, Klinikum Rechts der Isar der Technischen Universitär München, Ismaninger 22, 81675 Munich, Germany; dominik.weidlich@tum.de (D.W.); dimitrios.karampinos@tum.de (D.C.K.); 4 VAMPIRE Project, Computing (SSEN), University of Dundee, Nethergate, Dundee DD1 4HN, UK; mrk2k2@gmail.com; 5 Pillar of Engineering Product Development, Singapore University of Technology and Design, 8 Somapah Road, Singapore 487372, Singapore; subburaj@sutd.edu.sg

**Keywords:** magnetic resonance imaging, texture analysis, proton density fat fraction, paraspinal muscles, muscle strength

## Abstract

Texture analysis (TA) has shown promise as a surrogate marker for tissue structure, based on conventional and quantitative MRI sequences. Chemical-shift-encoding-based MRI (CSE-MRI)-derived proton density fat fraction (PDFF) of paraspinal muscles has been associated with various medical conditions including lumbar back pain (LBP) and neuromuscular diseases (NMD). Its application has been shown to improve the prediction of paraspinal muscle strength beyond muscle volume. Since mean PDFF values do not fully reflect muscle tissue structure, the purpose of our study was to investigate PDFF-based TA of paraspinal muscles as a predictor of muscle strength, as compared to mean PDFF. We performed 3T-MRI of the lumbar spine in 26 healthy subjects (age = 30 ± 6 years; 15 females) using a six-echo 3D spoiled gradient echo sequence for chemical-shift-encoding-based water–fat separation. Erector spinae (ES) and psoas (PS) muscles were segmented bilaterally from level L2–L5 to extract mean PDFF and texture features. Muscle flexion and extension strength was measured with an isokinetic dynamometer. Out of the eleven texture features extracted for each muscle, Kurtosis(global) of ES showed the highest significant correlation (*r* = 0.59, *p* = 0.001) with extension strength and Variance(global) of PS showed the highest significant correlation (*r* = 0.63, *p* = 0.001) with flexion strength. Using multivariate linear regression models, Kurtosis(global) of ES and BMI were identified as significant predictors of extension strength (R^2^_adj_ = 0.42; *p* < 0.001), and Variance(global) and Skewness(global) of PS were identified as significant predictors of flexion strength (R^2^_adj_ = 0.59; *p* = 0.001), while mean PDFF was not identified as a significant predictor. TA of CSE-MRI-based PDFF maps improves the prediction of paraspinal muscle strength beyond mean PDFF, potentially reflecting the ability to quantify the pattern of muscular fat infiltration. In the future, this may help to improve the pathophysiological understanding, diagnosis, monitoring and treatment evaluation of diseases with paraspinal muscle involvement, e.g., NMD and LBP.

## 1. Introduction

The paraspinal muscles constitute important muscles for the stability, movement and functionality of the lumbar spine [1]. Changes in paraspinal muscle volume and composition have been shown to be influenced by multiple demographic and anatomical factors: lumbar paravertebral muscle fat content is reported to be negatively associated with aging and positively associated with body mass index (BMI), as assessed by CT-based muscle density [2] and chemical-shift-encoding-based water–fat magnetic resonance imaging (CSE-MRI) [3,4]. Furthermore, these studies found that women are more susceptible to age-related changes than men, and paraspinal muscles are more susceptible to age-related changes than leg muscles.

The relationship between exercise and MRI characteristics reflecting muscle volume and composition have also been investigated previously. Early studies demonstrated increased transverse relaxation times (T2) after muscle exercise in lower leg [5], quadriceps [6,7] and ischiocrural muscles [8].

More importantly from a clinical perspective, a variety of medical conditions have been investigated in the context of muscle volume and composition. Degenerative disc disease and facet joint disease were shown to be positively associated with atrophy [9] and fatty infiltration [10] of skeletal muscle. Measurements of paraspinal muscle fat infiltration (MFI) were shown to agree well between CSE-MRI and magnetic resonance spectroscopy (MRS) [11], and to be strongly correlated with lumbar back pain (LBP) [12,13]. Regarding metabolic disorders, Karampinos et al. showed significant changes in the distribution of regional intermuscular adipose tissue distribution in patients suffering from type-2 diabetes mellitus [14]. A considerable amount of clinical research regarding changes in muscle volume and composition has focused on neuromuscular diseases (NMD), demonstrating an increased fat fraction, eventually resulting in extensive fatty replacement of paraspinal muscle tissue [15,16]. These results, based on quantitative CT and MRI, could have the potential to improve early diagnosis and monitoring of NMD patients, and, in the future, provide a valuable tool to evaluate the efficacy of NMD treatments. However, advanced quantitative imaging still has to find its way into the routine clinical management of NMD patients.

As a tool for treatment evaluation, quantitative muscle imaging has already been used successfully in patients with irreversible spinal cord injury, where an increase in thigh muscle cross-sectional area (CSA) was demonstrated after electrical stimulation therapy [17], exploiting the capability of 3D CT image segmentation to capture clinically relevant changes in muscle size and quality [18].

The described relationships with demographic factors, exercise and the mentioned medical conditions prove that imaging-based quantification of skeletal muscle has been extensively investigated in the past, but still needs further development to become a viable clinical tool. Therefore, there is an increasing interest in paraspinal muscle structure as a potential prognostic and diagnostic marker for spine and muscle health, in addition to muscle volume and composition.

Magnetic resonance imaging (MRI) constitutes a non-invasive method for the qualitative and quantitative characterization of muscle tissue. Among other things, it has been applied to assess volume, MFI, and inflammation of muscle tissue. Measurement of the proton density fat fraction (PDFF) by CSE-MRI has been shown to be robust and reliable, validated with magnetic resonance spectroscopy (MRS) [11] and histology [19] and therefore evolved as arguably the most promising imaging technique for the assessment of MFI. However, mean PDFF ignores the variability in muscular structure and distribution of muscle fat and may therefore not fully reflect muscle quality.

Texture analysis (TA) has emerged as an advanced analysis method in order to extract more quantitative information contained in medical imaging data [20,21]. It has been used in a variety of applications including neurologic and oncologic imaging [22,23,24,25].

So far, TA in musculoskeletal applications has mainly been based on non-quantitative imaging data including sonography [26] and computed tomography [27]. Regarding MRI, conventional T2-weighted sequences have been used for TA in the context of lumbar spinal stenosis (LSS) [28,29], and recently, Burian et al. demonstrated the feasibility of TA based on CSE-MRI-derived PDFF maps in vertebral bone marrow [30].

Investigations, relating quantitative CT and MRI to strength measurements of skeletal muscle, have been performed before. Recenti et al. extracted soft tissue parameters from mid-femur CT scans to construct a machine leaning system which could predict isometric leg strength [31]. Sinha et al. showed that four weeks of chronic unloading resulted in significant atrophy reflected by CSA reduction of calf muscle, which could only partially explain the observed reduction in muscle strength. Their results suggest that MRI-based strain rate indices may provide additional determinants of muscle force loss independent of muscle mass. Furthermore, the association of mean PDFF with strength measurements has been investigated in healthy volunteers in paraspinal and thigh muscles where mean PDFF has been shown to be a superior predictor of muscle strength compared to CSA [32,33]. In conjunction with previous findings, demonstrating an association of increased paraspinal MFI and decreased muscle function [1,34,35], this is another indicator that muscle quality is a significant determinator of muscle function in addition to muscle mass. The quality of muscular tissue is not only affected by its composition, but also by its structure. However, muscle structure is not sufficiently represented by mean PDFF and its association with paraspinal muscle function has not been studied before. In general, TA of muscle tissue can be performed on non-quantitative as well as quantitative MRI data [28,30]. However, TA based on PDFF maps can potentially yield information about muscular fat distribution beyond mere fat content, thus enabling the differentiation of muscles with different patterns of fat infiltration.

Therefore, the aim of the present study was to investigate whether quantification of paraspinal muscle fat distribution improves the prediction of muscle strength beyond mean PDFF. For this purpose, we analyzed the association between texture features of CSE-MRI-derived PDFF maps with isometric strength, measured with an isokinetic dynamometer, in healthy subjects.

## 2. Materials and Methods

### 2.1. Subjects

In total, 26 healthy subjects (15 women, 11 men; age = 30.27 ± 6.12 years, range: 21–42 years; BMI = 27.01 ± 2.69 kg/m^2^, range: 22.16–32.40 kg/m^2^) were recruited for this study as outlined previously [32,33]. Inclusion and exclusion criteria are summarized in Table 1.

Informed written consent was obtained from all subjects for MRI examination and biometrical strength measurements. The study protocol was in accordance with the Declaration of Helsinki and its later amendments and was approved by the local institutional review board (‘Ethikkommission der TU München’; date of approval: 12 December 2015; file number 482/15S).

### 2.2. MR Imaging

All subjects underwent MRI on the same 3T system (Ingenia, Philips Healthcare, Best, The Netherlands) using the built-in 12-channel posterior coil and a 16-channel anterior coil placed upon the abdomen. Subjects were positioned head-first in a supine position. An axially prescribed six-echo 3D spoiled gradient echo sequence was used for chemical shift encoding-based water–fat separation covering the lumbar spine. The sequence acquired the six echoes in a single TR using non-flyback (bipolar) read-out gradients with the following imaging parameters: TR/TEmin/ΔTE = 6.4/1.1/0.8 ms, field of view (FOV) = 220 × 401 × 252 mm^3^ (AP × LR × SI), voxel size = 3.2 × 2.0 × 4.0 mm^3^, frequency encoding direction = LR, no SENSE, scan time = 1 min 25 s. A saturation slab with a thickness of 80 mm was placed anterior to the FOV to minimize artifacts from breathing motion. A flip angle of 3° was used to minimize T_1_-bias effects [38]. The gradient echo imaging data was processed online using the vendor’s routines as described here: The multi-echo mDIXON algorithm performs a phase error correction followed by a complex-based water–fat decomposition using a pre-calibrated seven-peak fat spectrum and a single T_2_* to model the signal variation with echo time. The imaging-based PDFF maps were computed as the ratio of the fat signal over the sum of fat and water signals.

### 2.3. MR Image Segmentation

**Figure 1:**
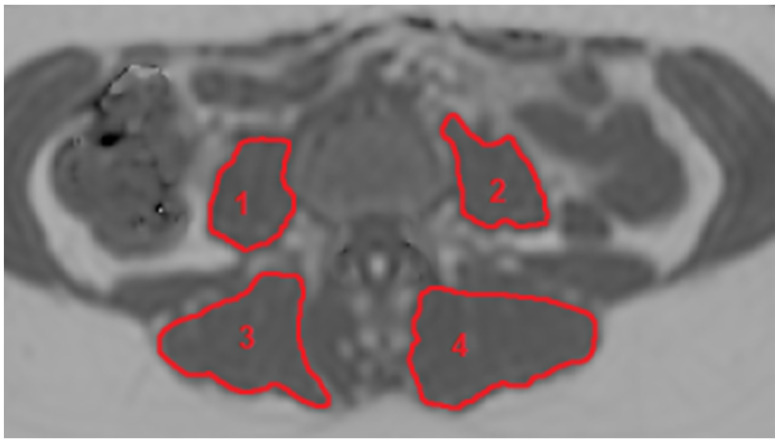
Representative axial PDFF map showing the ROIs of the four manually segmented muscles. 1: right psoas, 2: left psoas, 3: right erector spinae, 4: left erector spinae. (PDFF, proton density fat fraction; ROI, region of interest).

Segmentation of the paraspinal muscles was performed by drawing regions of interest (ROIs) on each slice of the PDFF maps using the open-source software MITK (Medical Imaging Interaction Toolkit, German Cancer Research Center, Division of Medical and Biological Informatics, Heidelberg, Germany) by a radiologist, resulting in three-dimensional segmentation masks. Right and left erector spinae muscles (ES) as well as right and left psoas muscles (PS) were segmented separately from the upper endplate level of L2 to the lower endplate level of L5 as reported previously [32]. ROIs were placed at the muscle contour to minimize the inclusion of subcutaneous fat or the muscle–fat interface. A representative axial slice of a PDFF map with corresponding segmentation masks of ES and PS muscles is shown in Figure 1. Mean PDFF of each of the four muscles was extracted. For both muscle groups (ES and PS), right and left mean PDFF were averaged and weighted by the respective muscle volumes to obtain bilateral mean PDFF values (PDFF_ES_, PDFF_PS_).

### 2.4. Texture Analysis of PDFF Maps

Texture analysis was performed on the PDFF maps of the segmented paraspinal muscles. Three global features (variance, skewness, kurtosis) and the following eight second-order features were extracted: energy, entropy, contrast, homogeneity, and correlation were calculated according to [39], variance and sum-average according to [40] and dissimilarity according to [41]. All texture features were calculated for each of the four muscles. Analogously to mean PDFF values, for both muscle groups (ES and PS), values were averaged over both sides, weighted by the respective muscle volumes to obtain bilateral texture feature values (e.g., Variance(global)_ES_, Variance(global)_PS_).

Global features were extracted from intensity histograms. In histogram analysis, there is no universal method for choosing the ideal number and size of bins. The number of bins used in our analysis was calculated by taking the median of three different methods, known as Sturges’ method, Scott’s method and the Freedman–Diaconis method since this yielded the most reasonable results compared to visual inspection of the histograms and showed the best representation of the relevant data characteristics [42,43,44].

Second-order features were extracted using gray-level GLCM analysis [39]. As a preprocessing step, gray level quantization of the PDFF maps was performed to prevent sparseness by normalizing the image intensities using 200 equally sized bins and the minimum and maximum gray levels present, corresponding to values of 0% and 100%, respectively.

GLCM was obtained by computing the joint probability of two adjacent voxel intensities at a given offset *d* = (*dx*, *dy*, *dz*) and angular directions *θ* = (0°, 45°, 90°, and 135°). *dx, dy* and *dz* denote the displacement along the *x*-, *y*- and *z*-axis, respectively.

For 3D-GLCM analysis, the co-occurrence probabilities of voxel intensities were computed from the 26 neighbors, aligned in 13 directions taking into account discretization length differences. The mean value of the features computed from the 13 directions ensures the rotation invariance. Image preprocessing, including isotropic resampling, gray level uniform quantization and texture analysis were performed using MATLAB 2018 (MathWorks Inc., Natick, MA, USA) and a radiomics toolbox (https://github.com/mvallieres/radiomics/) [45,46,47].

### 2.5. Isometric Muscle Strength Measurements

Isometric muscle strength measurements of back extensors and flexors were performed on two separate visits using an isokinetic rotational dynamometer (IsoMed Back Module, D&R Ferstl GmbH, Hemau, Germany) [32]. On the first visit, the objective was to familiarize the subject with the measurement procedure and train maximum isometric strength activation. Five to eight repeated measurements at maximum voluntary isometric contraction (MVIC) with 3 min breaks in-between were conducted. On the second visit, for each direction of motion (flexion and extension), the actual MVIC was acquired for data analysis as the maximum isometric torque [Nm] of three consecutive measurements with 3 min of recovery in-between. Subjects were instructed to be fully recovered for the actual measurement (no physical activity for two days before these visits). On both dates, the measurements were preceded by a standardized warm-up.

### 2.6. Statistical Analysis

Statistical analyses were performed with SPSS 26.0 (SPSS Inc., Chicago, IL, USA) using a two-sided level of significance *α* = 0.05 for all statistical tests.

The Kolmogorov–Smirnov test indicated normally distributed data for the majority of parameters. Mean and standard deviation (SD) of age, BMI, PDFF_ES_, PDFF_PS_ and texture features were calculated for males and females and sex-dependent differences were compared using unpaired t-tests. Pearson correlation coefficient *r* was calculated and Bonferroni correction (corrected level of significance *α_corr_* = *α*/24 = 0.0021) was applied to identify sex-dependent differences of texture features as well as significant correlations of PDFF_ES_, PDFF_PS_ and texture features vs. extension strength and flexion strength, respectively.

Stepwise multivariate linear regression models were used to determine significant predictors of extension and flexion strength. Independent variables were age, BMI, PDFF_ES_, PDFF_PS_ and the eleven texture features for ES and PS, respectively, resulting in 26 potential predicting variables. Inclusion (*p* < 0.05) and exclusion (*p* > 0.10) of independent variables in the linear regression models were based on the *p*-values of the F-test. Adjusted regression coefficients (R^2^_adj_) were calculated for each model.

## 3. Results

In Table 2, mean ± SD of age, BMI, PDFF_ES_, PDFF_PS_ and the 22 calculated texture features (11 for each muscle group), grouped by sex, are displayed. Age and BMI showed no significant difference between females and males (*p* > 0.05). Both PDFF_ES_ and PDFF_PS_ were higher in females compared to males, however the difference was only significant for PDFF_ES_ (*p* = 0.015). After adjustment for multiple comparisons, eight of the 22 texture features showed significant sex-dependent differences: Variance(global)_ES_, Skewness(global)_ES_, Variance(global)_PS_ and Homogeneity_PS_ were greater in male subjects, while Contrast_PS_, Entropy_PS_, Variance_PS,_ and Dissimilarity_PS_ were greater in female subjects.

**Figure 2:**
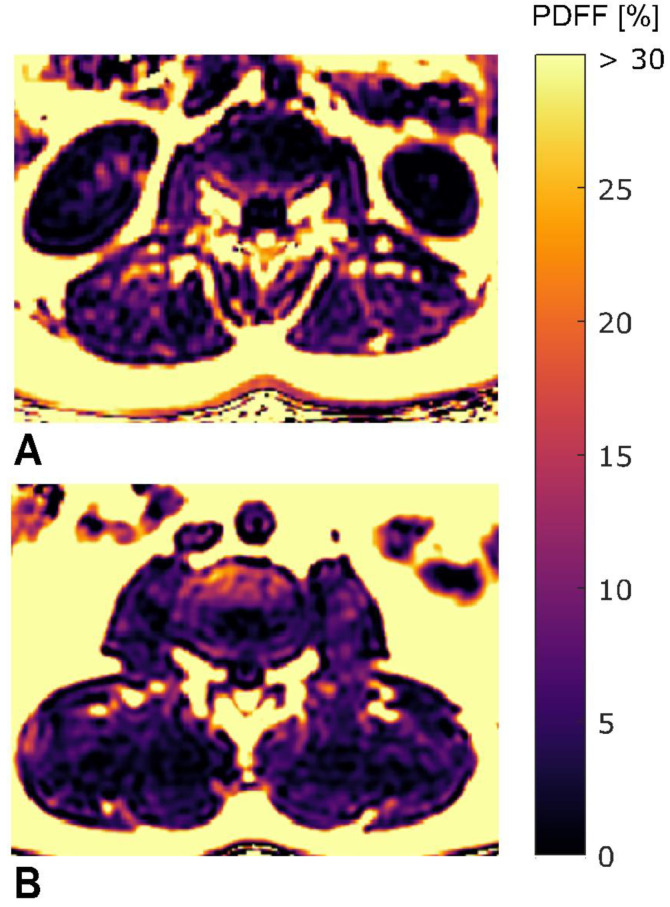
Sample color-coded axial PDFF maps of two study subjects. (**A**): subject with low strength (MVIC_ext_ = 103.6 Nm, MVIC_flex_ = 112.5 Nm) and high mean PDFF values (PDFF_ES_ = 14.3%, PDFF_PS_ = 5.6%). (**B**): subject with high strength (MVIC_ext_ = 335.9 Nm, MVIC_flex_ = 255.6 Nm) and low mean PDFF values (PDFF_ES_ = 8.3%, PDFF_PS_ = 3.9%). The upper limit of the color window was set to 30% to better depict the PDFF values within the paraspinal muscles. (PDFF_ES/PS_, proton density fat fraction of erector spinae and psoas muscles; MVIC_ext/flex_, maximum voluntary isometric contraction of extension and flexion; Nm, newton meter).

Representative color-coded PDFF maps are shown in Figure 2. PDFF_ES_ and PDFF_PS_ showed no significant correlation with age (*p* = 0.991 for PDFF_ES_, *p* = 0.594 for PDFF_PS_) or BMI (*p* = 0.345 for PDFF_ES_, *p* = 0.456 for PDFF_PS_). PDFF_ES_ showed no significant correlation with PDFF_PS_ (*p* = 0.221). Extension strength correlated with flexion strength (*r* = 0.62; *p* < 0.001).

**Figure 3:**
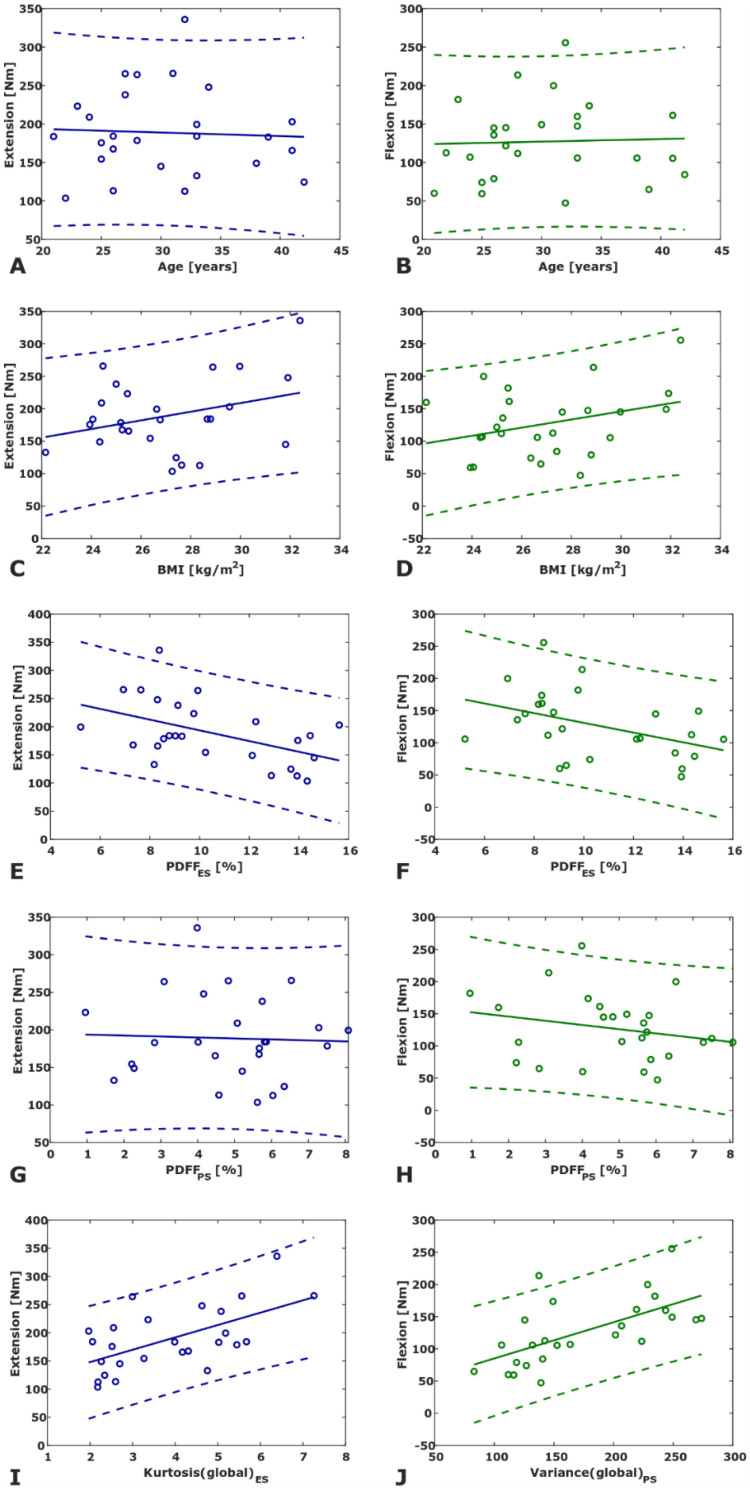
Scatter plots of age (**A**,**B**), BMI (**C**,**D**), erector spinae mean PDFF (**E**,**F**), psoas mean PDFF (**G**,**H**) vs. maximum extension torque (left column, blue) and maximum flexion torque (right column, green). The bottom row displays the texture feature exhibiting the highest correlation with extension strength (**I**) and flexion strength (**J**), respectively. (PDFF, proton density fat fraction).

Four of the 24 analyzed variables (PDFF_ES_, PDFF_PS_, and 22 texture features) showed significant correlations with strength measurements. Kurtosis(global)_ES_ showed the highest significant correlation (*r* = 0.59, *p* = 0.001) with extension strength and Variance(global)_PS_ showed the highest significant correlation (*r* = 0.63, *p* < 0.001) with flexion strength (Supplementary Material Table S1). PDFF_ES_ and extension strength showed the highest correlation of mean PDFF and strength measurements, however not significant after adjustment for multiple comparisons (*r* = −0.491, *p* = 0.011; *α_corr_* = 0.05/24 = 0.0021). Scatter plots of age, BMI, PDFF_ES_, PDFF_PS_, and texture feature vs. extension strength and flexion strength, respectively, are shown in Figure 3.

In the multivariate linear regression analyses (independent variables: age, BMI, PDFF_ES_, PDFF_PS_, texture features of ES and PS), Kurtosis(global)_ES_ (*p* = 0.001) and BMI (*p* = 0.042) were identified as statistically significant predictors of extension strength (R^2^_adj_ = 0.42; *p* = 0.001), and Variance(global)_PS_ (*p* < 0.001) and Skewness(global)_PS_ (*p* = 0.001) were identified as statistically significant predictors of flexion strength (R^2^_adj_ = 0.59; *p* < 0.001). Of note, in these models, which predicted extension and flexion strength best, PDFF_ES_ and PDFF_PS_ were not identified as significant contributors. Age was not identified as a statistically significant confounder in any of the models.

## 4. Discussions

In the present study, we demonstrated that paraspinal muscle texture features, extracted from CSE-MRI-derived PDFF maps, significantly correlated with muscle strength. Texture features better predicted paraspinal muscle strength than mean PDFF alone.

We observed significant sex-dependent differences for several of the extracted texture features: Variance(global) was greater in males than females for both psoas and erector spinae muscles. Current literature results have shown sex-dependent differences in muscle cross sectional areas, muscle strength, and fatty muscle infiltration [3,4,48]. However, to date, no comparable studies have reported on the sex-dependence of MR-based texture features of paraspinal muscles and future studies are needed to confirm these initial results.

The use of texture features for quantitative medical imaging analysis has been increasing in recent years, particularly in oncological imaging. Furthermore, Burian et al. performed TA of vertebral bone marrow PDFF maps, demonstrating its feasibility and ability to differentiate pre- from postmenopausal women equally well as mean PDFF [30]. In particular, the authors identified the texture features Contrast and Dissimilarity as best discriminators. In contrast to the application in bone marrow, we performed PDFF-based TA of muscle tissue. In comparison to bone marrow, muscle tissue has remarkably lower fat content and higher heterogeneity of fat distribution. This potentially explains why the mean values of the texture features Variance and Variance(global) are remarkably higher in the study of Burian et al. as compared to our study in paraspinal muscles.

To the best of our knowledge, preliminary work on TA in this anatomical region is very scarce. In the context of lumbar spinal stenosis (LSS), TA was shown to be a reproducible tool with the potential to improve LSS detection compared to qualitative assessment [28,29,49]. In our study, we performed paraspinal muscle TA on CSE-MRI-derived PDFF maps, a fast-imaging technique exhibiting good reproducibility [32]. Compared to semi-quantitative analysis of conventional T2-weighted sequences, PDFF extraction based on CSE-MRI offers a more reliable and comparable approach for the assessment of water–fat composition. Both the T2-weighted approach and the CSE-MRI approach are sensitive to muscular fat infiltration. However, the T2-weighted approach is limited in the presence of other T2w-hyprintense alterations, e.g., muscular edema which occurs in NMD and other inflammatory conditions of the muscle.

The association of muscle composition and isokinetic strength measurements has been investigated before in paraspinal and thigh muscles [32,33]. In these studies, the authors showed that CSE-MRI-derived mean PDFF measurements of erector spinae, quadriceps and ischiocrural muscles improve the prediction of strength measurements beyond CSA, indicating that, in addition to muscle mass, composition of contractile muscle has a major effect on its function.

**Figure 4:**
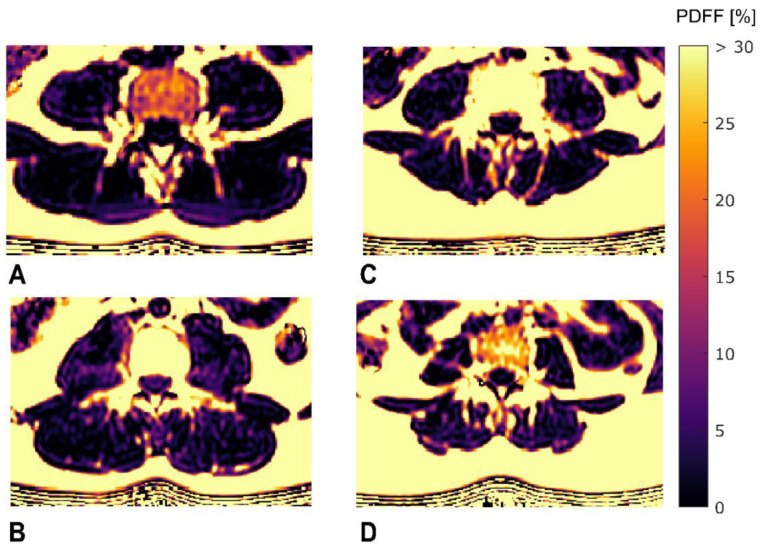
Sample color-coded axial PDFF maps of four study subjects visualizing inter-individual differences in texture parameters. The upper limit of the color window was set to 30% to better depict the PDFF values within the paraspinal muscles. (**A**,**B**): two subjects with comparable mean PDFF_ES_ (PDFF_ES_ = 6.94 and 7.33) and large difference in Kurtosis(global)_ES_ (Kurtosis(global)_ES_ = 7.257 and 4.309). Note the existence of more outliers at both ends of the PDFF spectrum within the erector spinae muscles in (**A**) reflected by a higher Kurtosis(global)_ES_ as compared to (**B**). (**C**,**D**): two subjects with comparable mean PDFF_PS_ (PDFF_PS_ = 5.81 and 5.67) and large difference in Variance(global)_PS_ (Variance (global)_PS_ = 273.76 and 116.31). Note the more prominent fatty streaks within the psoas muscles in (**C**) resulting from a more heterogeneous fat distribution and reflected by a higher Variance(global)_PS_ as compared to (**D**). ES, erector spinae muscles; PS, psoas muscles; PDFF_ES/PS_, proton density fat fraction of ES and PS.

In the present study, multivariate linear regression analysis identified certain texture features and BMI, but not mean PDFF, as significant predictors of extension and flexion strength. The identified first-order features increase with increasing variation in PDFF across the ROI and are particularly sensitive to extreme values relative to the mean. Those outliers are more likely to be encountered in the presence of localized muscular and perimuscular alterations, such as fat streaks, circumscribed edema, scarring and connective tissue proliferation. Hence, our results suggest that muscle structure as well as the pattern of MFI have a relevant impact on paraspinal muscle function and support the hypothesis that TA of muscle tissue based on PDFF maps can quantify the distribution of MFI. Put more figuratively, texture feature may better differentiate muscles with a rather homogeneous fat infiltration from muscles with fat streaks which have a more heterogeneous fat infiltration, although both have the same mean fat content. This is exemplified visually in Figure 4 which displays PDFF maps of subjects with comparable mean PDFF but a large difference in the texture feature exhibiting the highest correlation with extension strength (Figure 4A,B), and a large difference in the texture feature exhibiting the highest correlation with flexion strength (Figure 4C,D), respectively.

Diffusion tensor imaging (DTI) represents another MRI technique for quantitative assessment of tissue structure. Extension-to-flexion strength ratio of paraspinal muscles was shown to be best predicted by DTI parameters as compared to CSA and mean PDFF [50]. To some extent, these results can be considered to be in line with our findings, supporting the hypothesis that changes in paraspinal muscle function are related to structural alterations, such as the pattern of muscular fat infiltration.

The improved prediction of muscle strength by TA compared to mean PDFF is a very promising finding. The present study, which is the first to perform CSE-MRI-based TA of paraspinal muscles, supports the hypothesis that the pattern of MFI has a significant effect on muscular function and indicates that advanced postprocessing could reveal new insights into muscle structure and function in a time-efficient manner without the need for additional data acquisition. The use of quantitative PDFF maps instead of non-quantitative T2-weighted images is particularly appealing for the assessment of NMD, inflammatory muscle diseases, and other conditions presenting with muscular edema as explained above. Depending on the application, our approach may therefore serve as an analysis technique to replace or complement established parameters, such as CSA and PDFF, for the evaluation of aforementioned muscle conditions.

The present study is not without limitations. First, TA was performed on manually segmented ROIs of erector spinae muscles. Since the respective muscles were segmented as a whole, both intra- and intermuscular fat contributed to the PDFF_ES_ distribution and the subsequent TA. Second, only young healthy subjects with relatively low muscle mean PDFF were included resulting in a rather low range and narrow distribution of PDFF values. However, regardless of the low variance in muscle fat content, significant correlations of texture features and strength measurements could be observed suggesting that small changes in water–fat composition of the muscle are accompanied by structural changes that have a relevant effect on the biomechanical function of the muscle. To further characterize paraspinal muscle TA, particularly in a larger range of PDFF values, future studies are needed. Those studies should include subjects with a greater age range as well as patients suffering from conditions affecting muscle composition and structure, such as NMD or LBP. A longitudinal study could also reveal training effects on muscle structure and function by examining subjects before and after exercise intervention.

## 5. Conclusions

We showed that TA of CSE-MRI-based PDFF maps is feasible in paraspinal muscles. Our initial results in this anatomic region demonstrate improved prediction of muscle strength beyond mean PDFF, indicating that muscular function is related to muscle fat distribution. TA of CSE-MRI-based PDFF maps thus has the potential to differentiate muscles based on the pattern of MFI. As a consequence, it should be applied in relevant patient groups, since it could reveal new pathophysiological insights and help to improve diagnosis, monitoring, and treatment evaluation of certain medical conditions, such as NMD and LBP.

## Figures and Tables

**Table 1:** Inclusion and exclusion criteria of the 26 recruited subjects; IPAQ-sf, International Physical Activity Questionnaire—short form [36,37].

**Inclusion Criteria**	Age: 20–45 years BMI: 20–33 kg/m^2^ Completion of the IPAQ-sf with a score referring to a moderate level of physical activity (600–1500 metabolic equivalent of task-min/week)
**Exclusion Criteria**	Vertebral fractures Severe anatomical or pathological alterations of the spine (e.g., scoliosis, spondylolisthesis, degenerative disc disease, facet joint arthrosis) Neuromuscular disease Metabolic disease (e.g., diabetes mellitus) History of high-performance sports General MRI contraindications (e.g., cochlear implant, severe claustrophobia)

**Table 2:** Mean and standard deviation (mean ± SD) of subject characteristics (age and BMI), proton density fat fraction, and analyzed texture parameters, separately for erector spinae (ES) and psoas (PS) muscles and grouped by sex (male, n = 11; female, n = 15); *p*, *p*-value of unpaired *t*-test comparing male and female subjects.

	Male	Female	Total	*p*
Age [years]	30.73 ± 4.82	29.93 ± 7.07	30.27 ± 6.12	0.751
BMI [kg/m^2^]	27.86 ± 3.48	26.38 ± 1.82	27.01 ± 2.69	0.171
PDFF_ES_ [%]	8.93 ± 2.10	11.65 ± 2.92	10.50 ± 2.90	0.015
PDFF_PS_ [%]	4.22 ± 1.72	5.27 ± 1.81	4.83 ± 1.82	0.148
Variance(global)_ES_	335.72 ± 39.32	285.96 ± 25.84	307.01 ± 40.26	0.001
Skewness(global)_ES_	2.02 ± 0.20	1.71 ± 0.20	1.84 ± 0.25	0.001
Kurtosis(global)_ES_	4.71 ± 1.42	3.24 ± 1.31	3.86 ± 1.52	0.012
Energy_ES_ [×10^2^]	0.51 ± 0.26	0.29 ± 0.13	0.38 ± 0.22	0.008
Contrast_ES_	84.08 ± 26.92	110.65 ± 27.09	99.41 ± 29.67	0.021
Entropy_ES_	9.68 ± 0.63	10.36 ± 0.57	10.07 ± 0.68	0.008
Homogeneity_ES_	0.38 ± 0.04	0.33 ± 0.04	0.35 ± 0.05	0.007
Correlation_ES_	0.86 ± 0.02	0.87 ± 0.02	0.86 ± 0.02	0.553
SumAverage_ES_ [×10^2^]	0.18 ± 0.02	0.18 ± 0.02	0.18 ± 0.02	0.322
Variance_ES_ [×10^2^]	0.77 ± 0.25	1.08 ± 0.35	0.95 ± 0.35	0.021
Dissimilarity_ES_	5.10 ± 1.01	6.22 ± 1.02	5.75 ± 1.15	0.010
Variance(global)_PS_	223.66 ± 44.36	138.94 ± 36.00	174.79 ± 57.75	<0.001
Skewness(global)_PS_	1.15 ± 0.68	1.07 ± 0.47	1.11 ± 0.56	0.728
Kurtosis(global)_PS_	5.90 ± 1.98	4.22 ± 1.09	4.93 ± 1.72	0.011
Energy_PS_ [×10^2^]	0.19 ± 0.05	0.13 ± 0.04	0.16 ± 0.05	0.003
Contrast_PS_	104.98 ± 18.23	140.31 ± 27.83	125.36 ± 29.72	0.001
Entropy_PS_	10.28 ± 0.30	10.79 ± 0.29	10.57 ± 0.39	<0.001
Homogeneity_PS_	0.31 ± 0.02	0.28 ± 0.02	0.29 ± 0.03	<0.001
Correlation_PS_	0.73 ± 0.03	0.73 ± 0.04	0.73 ± 0.04	0.945
SumAverage_PS_ [×10^2^]	0.19 ± 0.02	0.20 ± 0.01	0.19 ± 0.01	0.187
Variance_PS_ [×10^2^]	0.48 ± 0.06	0.65 ± 0.12	0.58 ± 0.12	<0.001
Dissimilarity_PS_	5.95 ± 0.57	7.22 ± 0.82	6.68 ± 0.96	<0.001

## Data Availability

The datasets generated during and/or analyzed during the current study are available from the corresponding author on reasonable request.

## Supplementary Materials

The following are available online at https://www.mdpi.com/2075-4418/11/2/239/s1, Figure S1: Color-coded PDFF maps (sample axial slices) all 15 female subjects. The upper limit of the color window was set to 30% to better depict the PDFF values of the paraspinal muscles (PDFF, proton density fat fraction). Figure S2: Color-coded PDFF maps (sample axial slices) all eleven male subjects. The upper limit of the color window was set to 30% to better depict the PDFF values of the paraspinal muscles (PDFF, proton density fat fraction). Table S1: Pearson correlation coefficient *r* and corresponding *p*-values *p* for mean PDFF and texture features vs. extension strength (center column), and *r* and *p* for mean PDFF and texture features vs. flexion strength (right column), respectively.

## Abbreviations

AP	anterior-posterior direction
BMI	body mass index
CSA	cross-sectional area
CSE-MRI	chemical shift encoding-based water-fat magnetic resonance imaging
DTI	diffusion tensor imaging
ES	erector spinae muscle
FOV	field of view
GLCM	gray-level co-occurrence matrix
IPACQ-SF	International Physical Activity Questionnaire Short-Form
L2	second lumbar vertebra
L5	fifth lumbar vertebra
LSS	lumbar spinal stenosis
LR	left-right direction
MFI	muscle fat infiltration
MITK	Medical Imaging Interaction Toolkit
MRI	magnetic resonance imaging
MRS	magnetic resonance spectroscopy
MVIC	maximum voluntary isometric contraction
MVIC_ext/flex_,	MVIC of extension
MVIC_ext/flex_,	MVIC of flexion
[Nm]	newton meter
PDFF	proton density fat fraction
PDFF_ES_	PDFF of erector spinae muscles
PDFF_PS_	PDFF of psoas muscles
PS	psoas muscle
*r*	Pearson correlation coefficient
R^2^_adj_	adjusted coefficient of determination
ROI	region of interest
SD	standard deviation
SENSE	sensitivity encoding
SI	superior-inferior direction
T	Tesla
T1	longitudinal relaxation time
T2	transverse relaxation time
T2*	effective transverse relaxation time
TA	texture analysis
TE	echo time
ΔTE	echo time step
TR	repetition time

## References

[1] Hicks G.E., Simonsick E.M., Harris T.B., Newman A.B., Weiner D.K., Nevitt M.A., Tylavsky F.A. (2005). Cross-sectional associations between trunk muscle composition, back pain, and physical function in the health, aging and body composition study. J. Gerontol. A Biol. Sci. Med. Sci..

[2] Kalichman L., Hodges P., Li L., Guermazi A., Hunter D.J. (2010). Changes in paraspinal muscles and their association with low back pain and spinal degeneration: CT study. Eur. Spine J..

[3] Crawford R.J., Filli L., Elliott J.M., Nanz D., Fischer M.A., Marcon M., Ulbrich E.J. (2016). Age- and Level-Dependence of Fatty Infiltration in Lumbar Paravertebral Muscles of Healthy Volunteers. AJNR Am. J. Neuroradiol..

[4] Dahlqvist J.R., Vissing C.R., Hedermann G., Thomsen C., Vissing J. (2017). Fat Replacement of Paraspinal Muscles with Aging in Healthy Adults. Med. Sci. Sports Exerc..

[5] Fisher M.J., Meyer R.A., Adams G.R., Foley J.M., Potchen E.J. (1990). Direct relationship between proton T2 and exercise intensity in skeletal muscle MR images. Invest. Radiol..

[6] Shellock F.G., Fukunaga T., Mink J.H., Edgerton V.R. (1991). Acute effects of exercise on MR imaging of skeletal muscle: Concentric vs eccentric actions. AJR Am. J. Roentgenol..

[7] Takahashi H., Kuno S., Miyamoto T., Yoshioka H., Inaki M., Akima H., Katsuta S., Anno I., Itai Y. (1994). Changes in magnetic resonance images in human skeletal muscle after eccentric exercise. Eur. J. Appl. Physiol. Occup. Physiol..

[8] Mendez-Villanueva A., Suarez-Arrones L., Rodas G., Fernandez-Gonzalo R., Tesch P., Linnehan R., Kreider R., Di Salvo V. (2016). MRI-Based Regional Muscle Use during Hamstring Strengthening Exercises in Elite Soccer Players. PLoS ONE.

[9] Sun D., Liu P., Cheng J., Ma Z., Liu J., Qin T. (2017). Correlation between intervertebral disc degeneration, paraspinal muscle atrophy, and lumbar facet joints degeneration in patients with lumbar disc herniation. BMC Musculoskelet Disord..

[10] Sebro R., O’Brien L., Torriani M., Bredella M.A. (2016). Assessment of trunk muscle density using CT and its association with degenerative disc and facet joint disease of the lumbar spine. Skeletal Radiol..

[11] Fischer M.A., Nanz D., Shimakawa A., Schirmer T., Guggenberger R., Chhabra A., Carrino J.A., Andreisek G. (2013). Quantification of muscle fat in patients with low back pain: Comparison of multi-echo MR imaging with single-voxel MR spectroscopy. Radiology.

[12] Kjaer P., Bendix T., Sorensen J.S., Korsholm L., Leboeuf-Yde C. (2007). Are MRI-defined fat infiltrations in the multifidus muscles associated with low back pain?. BMC Med..

[13] Teichtahl A.J., Urquhart D.M., Wang Y., Wluka A.E., Wijethilake P., O’Sullivan R., Cicuttini F.M. (2015). Fat infiltration of paraspinal muscles is associated with low back pain, disability, and structural abnormalities in community-based adults. Spine J..

[14] Karampinos D.C., Baum T., Nardo L., Alizai H., Yu H., Carballido-Gamio J., Yap S.P., Shimakawa A., Link T.M., Majumdar S. (2012). Characterization of the regional distribution of skeletal muscle adipose tissue in type 2 diabetes using chemical shift-based water/fat separation. J. Magn Reson Imaging.

[15] Hadar H., Gadoth N., Heifetz M. (1983). Fatty replacement of lower paraspinal muscles: Normal and neuromuscular disorders. AJR Am. J. Roentgenol..

[16] Dahlqvist J.R., Vissing C.R., Thomsen C., Vissing J. (2014). Severe paraspinal muscle involvement in facioscapulohumeral muscular dystrophy. Neurology.

[17] Kern H., Carraro U. (2020). Home-Based Functional Electrical Stimulation of Human Permanent Denervated Muscles: A Narrative Review on Diagnostics, Managements, Results and Byproducts Revisited 2020. Diagnostics.

[18] Edmunds K.J., Gislason M.K., Arnadottir I.D., Marcante A., Piccione F., Gargiulo P. (2016). Quantitative Computed Tomography and Image Analysis for Advanced Muscle Assessment. Eur. J. Transl. Myol..

[19] Smith A.C., Parrish T.B., Abbott R., Hoggarth M.A., Mendoza K., Chen Y.F., Elliott J.M. (2014). Muscle-fat MRI: 1.5 Tesla and 3.0 Tesla versus histology. Muscle Nerve.

[20] Gillies R.J., Kinahan P.E., Hricak H. (2016). Radiomics: Images Are More than Pictures, They Are Data. Radiology.

[21] Aerts H.J., Velazquez E.R., Leijenaar R.T., Parmar C., Grossmann P., Carvalho S., Bussink J., Monshouwer R., Haibe-Kains B., Rietveld D. (2014). Decoding tumour phenotype by noninvasive imaging using a quantitative radiomics approach. Nat. Commun..

[22] Hainc N., Stippich C., Stieltjes B., Leu S., Bink A. (2017). Experimental Texture Analysis in Glioblastoma: A Methodological Study. Invest. Radiol..

[23] Ingrisch M., Schneider M.J., Norenberg D., Negrao de Figueiredo G., Maier-Hein K., Suchorska B., Schuller U., Albert N., Bruckmann H., Reiser M. (2017). Radiomic Analysis Reveals Prognostic Information in T1-Weighted Baseline Magnetic Resonance Imaging in Patients With Glioblastoma. Invest. Radiol..

[24] Hwang I.P., Park C.M., Park S.J., Lee S.M., McAdams H.P., Jeon Y.K., Goo J.M. (2015). Persistent Pure Ground-Glass Nodules Larger Than 5 mm: Differentiation of Invasive Pulmonary Adenocarcinomas From Preinvasive Lesions or Minimally Invasive Adenocarcinomas Using Texture Analysis. Invest. Radiol..

[25] Pickles M.D., Lowry M., Gibbs P. (2016). Pretreatment Prognostic Value of Dynamic Contrast-Enhanced Magnetic Resonance Imaging Vascular, Texture, Shape, and Size Parameters Compared With Traditional Survival Indicators Obtained From Locally Advanced Breast Cancer Patients. Invest. Radiol..

[26] Sogawa K., Nodera H., Takamatsu N., Mori A., Yamazaki H., Shimatani Y., Izumi Y., Kaji R. (2017). Neurogenic and Myogenic Diseases: Quantitative Texture Analysis of Muscle US Data for Differentiation. Radiology.

[27] Mookiah M.R.K., Rohrmeier A., Dieckmeyer M., Mei K., Kopp F.K., Noel P.B., Kirschke J.S., Baum T., Subburaj K. (2018). Feasibility of opportunistic osteoporosis screening in routine contrast-enhanced multi detector computed tomography (MDCT) using texture analysis. Osteoporos Int..

[28] Mannil M., Burgstaller J.M., Thanabalasingam A., Winklhofer S., Betz M., Held U., Guggenberger R. (2018). Texture analysis of paraspinal musculature in MRI of the lumbar spine: Analysis of the lumbar stenosis outcome study (LSOS) data. Skeletal Radiol..

[29] Mannil M., Burgstaller J.M., Held U., Farshad M., Guggenberger R. (2019). Correlation of texture analysis of paraspinal musculature on MRI with different clinical endpoints: Lumbar Stenosis Outcome Study (LSOS). Eur. Radiol..

[30] Burian E., Subburaj K., Mookiah M.R.K., Rohrmeier A., Hedderich D.M., Dieckmeyer M., Diefenbach M.N., Ruschke S., Rummeny E.J., Zimmer C. (2019). Texture analysis of vertebral bone marrow using chemical shift encoding-based water-fat MRI: A feasibility study. Osteoporos Int..

[31] Recenti M., Ricciardi C., Edmunds K., Gislason M.K., Gargiulo P. (2020). Machine learning predictive system based upon radiodensitometric distributions from mid-thigh CT images. Eur. J. Transl. Myol..

[32] Schlaeger S., Inhuber S., Rohrmeier A., Dieckmeyer M., Freitag F., Klupp E., Weidlich D., Feuerriegel G., Kreuzpointner F., Schwirtz A. (2019). Association of paraspinal muscle water-fat MRI-based measurements with isometric strength measurements. Eur. Radiol..

[33] Inhuber S., Sollmann N., Schlaeger S., Dieckmeyer M., Burian E., Kohlmeyer C., Karampinos D.C., Kirschke J.S., Baum T., Kreuzpointner F. (2019). Associations of thigh muscle fat infiltration with isometric strength measurements based on chemical shift encoding-based water-fat magnetic resonance imaging. Eur. Radiol. Exp..

[34] Goodpaster B.H., Carlson C.L., Visser M., Kelley D.E., Scherzinger A., Harris T.B., Stamm E., Newman A.B. (2001). Attenuation of skeletal muscle and strength in the elderly: The Health ABC Study. J. Appl. Physiol..

[35] Goodpaster B.H., Park S.W., Harris T.B., Kritchevsky S.B., Nevitt M., Schwartz A.V., Simonsick E.M., Tylavsky F.A., Visser M., Newman A.B. (2006). The loss of skeletal muscle strength, mass, and quality in older adults: The health, aging and body composition study. J. Gerontol. A Biol. Sci. Med. Sci..

[36] Guedes D.P., Lopes C.C., Guedes J.E.R.P. (2005). Reprodutibilidade e validade do Questionário Internacional de Atividade Física em adolescentes. Rev. Bras. Med. Esporte.

[37] Kurtze N., Rangul V., Hustvedt B.E. (2008). Reliability and validity of the international physical activity questionnaire in the Nord-Trondelag health study (HUNT) population of men. BMC Med. Res. Methodol..

[38] Karampinos D.C., Yu H., Shimakawa A., Link T.M., Majumdar S. (2011). T(1)-corrected fat quantification using chemical shift-based water/fat separation: Application to skeletal muscle. Magn Reson Med..

[39] Haralick R.M., Shanmugam K., Dinstein I. (1973). Textural Features for Image Classification. IEEE Trans. Syst. Man Cybern..

[40] Assefa D., Keller H., Menard C., Laperriere N., Ferrari R.J., Yeung I. (2010). Robust texture features for response monitoring of glioblastoma multiforme on T1-weighted and T2-FLAIR MR images: A preliminary investigation in terms of identification and segmentation. Med. Phys.

[41] Thibault G., Devic C., Fertil B., Mari J., Sequeira J. (2007). Indices de formes: De la 2D vers la 3D-Application au classement de noyaux de cellules. Journées de l’Association Francophone d’Informatique Graphique.

[42] Freedman D. (1981). On the histogram as a density estimator: L2 theory. Probab Theory Relat Fields.

[43] Scott D.W. (1979). On optimal and data-based histograms. Biometrika.

[44] Sturges H.A. (1926). The choice of a class interval. J. Am. Stat. Assoc.

[45] Vallieres M., Freeman C.R., Skamene S.R., El Naqa I. (2015). A radiomics model from joint FDG-PET and MRI texture features for the prediction of lung metastases in soft-tissue sarcomas of the extremities. Phys. Med. Biol..

[46] Zhou H., Vallieres M., Bai H.X., Su C., Tang H., Oldridge D., Zhang Z., Xiao B., Liao W., Tao Y. (2017). MRI features predict survival and molecular markers in diffuse lower-grade gliomas. Neuro Oncol..

[47] Vallieres M., Kay-Rivest E., Perrin L.J., Liem X., Furstoss C., Aerts H., Khaouam N., Nguyen-Tan P.F., Wang C.S., Sultanem K. (2017). Radiomics strategies for risk assessment of tumour failure in head-and-neck cancer. Sci. Rep..

[48] Miller A.E., MacDougall J.D., Tarnopolsky M.A., Sale D.G. (1993). Gender differences in strength and muscle fiber characteristics. Eur. J. Appl. Physiol. Occup. Physiol..

[49] Huber F.A., Stutz S., Vittoria de Martini I., Mannil M., Becker A.S., Winklhofer S., Burgstaller J.M., Guggenberger R. (2019). Qualitative versus quantitative lumbar spinal stenosis grading by machine learning supported texture analysis-Experience from the LSOS study cohort. Eur. J. Radiol..

[50] Klupp E., Cervantes B., Schlaeger S., Inhuber S., Kreuzpointer F., Schwirtz A., Rohrmeier A., Dieckmeyer M., Hedderich D.M., Diefenbach M.N. (2019). Paraspinal Muscle DTI Metrics Predict Muscle Strength. J. Magn Reson Imaging.

