# Supplementary material for: Texture Features of Proton Density Fat Fraction Maps from Chemical Shift Encoding-Based MRI Predict Paraspinal Muscle Strength
Source: Diagnostics (Basel). 2021 Feb 4;11(2):239. doi: 10.3390/diagnostics11020239 (PMC7913879; doi:10.3390/diagnostics11020239)
Supplement: Supplementary file 1 [file diagnostics-11-00239-s001.pdf]

Supplementary Material

	TF vs. Extension strength		TF vs. Flexion strength	
	r	*p*	*r*	*p*
**PDFF$_{ES}$**	-0.49	0.011	-0.42	0.031
**PDFF$_{PS}$**	-0.04	0.839	-0.23	0.258
**Variance(global)$_{ES}$**	0.40	0.044	0.62	**0.001**
**Skewness(global)$_{ES}$**	0.47	0.015	0.54	0.004
**Kurtosis(global)$_{ES}$**	0.59	**0.001**	0.49	0.011
**Energy$_{ES}$**	0.19	0.346	0.28	0.160
**Contrast$_{ES}$**	-0.23	0.260	-0.37	0.064
**Entropy$_{ES}$**	-0.33	0.102	-0.35	0.075
**Homogeneity$_{ES}$**	0.33	0.098	0.37	0.063
**Correlation$_{ES}$**	-0.50	0.010	-0.17	0.405
**SumAverage$_{ES}$**	-0.43	0.028	-0.25	0.213
**Variance$_{ES}$**	-0.38	0.052	-0.40	0.040
**Dissimilarity$_{ES}$**	-0.31	0.120	-0.39	0.048
**Variance(global)$_{PS}$**	0.33	0.099	0.63	**0.001**
**Skewness(global)$_{PS}$**	-0.08	0.700	-0.18	0.381
**Kurtosis(global)$_{PS}$**	0.29	0.152	0.24	0.238
**Energy$_{PS}$**	0.36	0.068	0.36	0.071
**Contrast$_{PS}$**	-0.30	0.135	-0.55	0.004
**Entropy$_{PS}$**	-0.38	0.058	-0.48	0.014
**Homogeneity$_{PS}$**	0.41	0.036	0.52	0.006
**Correlation$_{PS}$**	0.04	0.865	0.06	0.771
**SumAverage$_{PS}$**	-0.08	0.689	0.06	0.775
**Variance$_{PS}$**	-0.28	0.172	-0.56	0.003
**Dissimilarity$_{PS}$**	-0.36	0.072	-0.58	**0.002**

Supplementary Table S1: Pearson correlation coefficient ***r*** and corresponding p-values ***p*** for mean PDFF and texture features vs. extension strength (center column), and ***r*** and ***p*** for mean PDFF and texture features vs. flexion strength (right column), respectively. Bold p-values indicate significant

correlation after Bonferroni correction for multiple comparisons (Bonferroni-corrected level of significance $\alpha_{corr} = \alpha/n_{tf} = 0.05/24 = 0.0021$). TF, texture feature; n_{tf}, number of analyzed texture features; $PDFF_{ES}$, mean PDFF of erector spinae muscle; $PDFF_{PS}$, mean PDFF of psoas muscle; ***r***, Pearson correlation coefficient; ***p***, corresponding p-value.

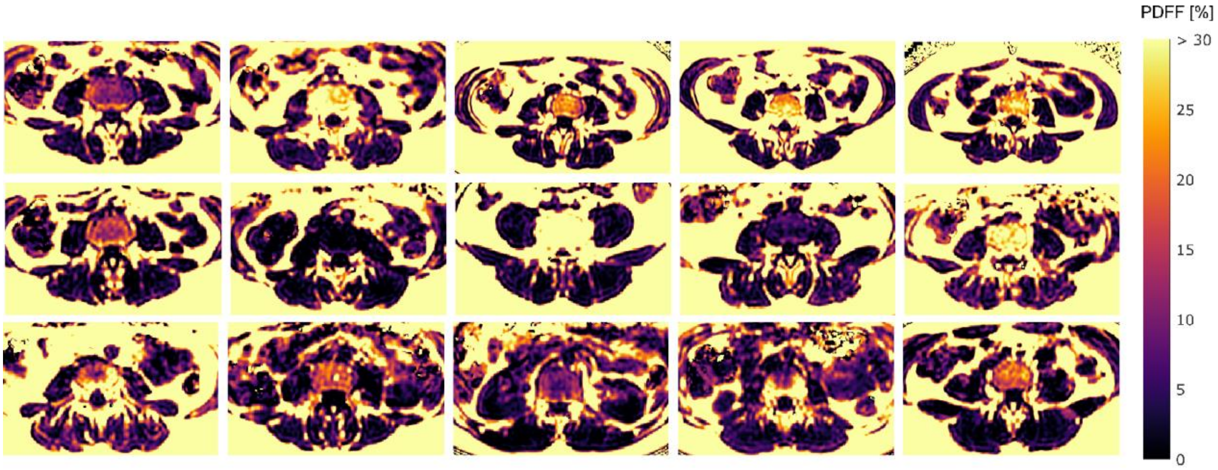

Supplementary Figure S1

Color-coded PDFF maps (sample axial slices) all 15 female subjects. The upper limit of the color window was set to 30 % to better depict the PDFF values of the paraspinal muscles (PDFF, proton density fat fraction).

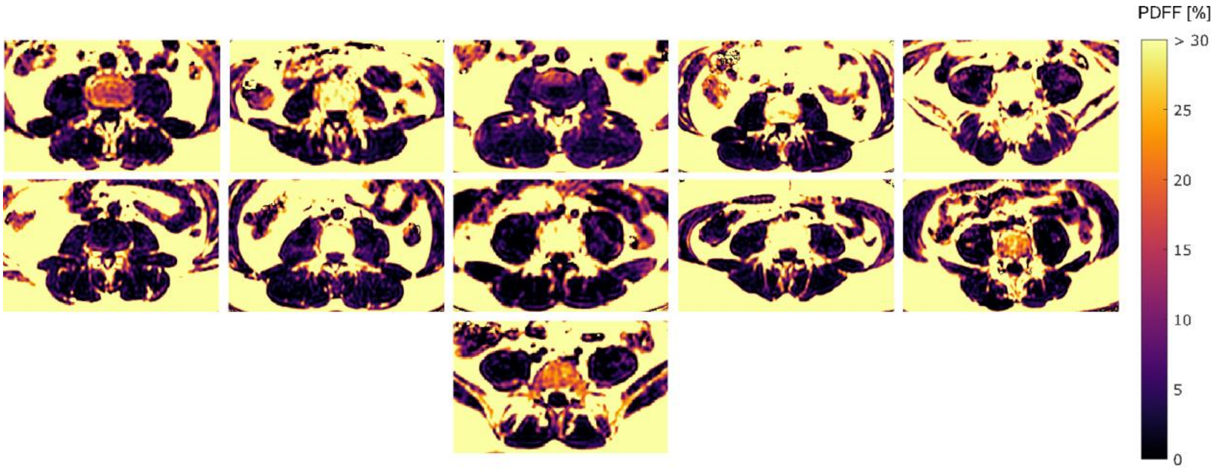

Supplementary Figure S2

Color-coded PDFF maps (sample axial slices) all eleven male subjects. The upper limit of the color window was set to 30 % to better depict the PDFF values of the paraspinal muscles (PDFF, proton density fat fraction).
